# Chemically Modified Peptide Scaffolds Target the CFTR-Associated Ligand PDZ Domain

**DOI:** 10.1371/journal.pone.0103650

**Published:** 2014-08-19

**Authors:** Jeanine F. Amacher, Ruizhi Zhao, Mark R. Spaller, Dean R. Madden

**Affiliations:** 1 Department of Biochemistry, Geisel School of Medicine at Dartmouth, Hanover, New Hampshire, United States of America; 2 Department of Chemistry, Dartmouth College, Hanover, New Hampshire, United States of America; 3 Norris Cotton Cancer Center, Dartmouth-Hitchcock Medical Center, Lebanon, New Hampshire, United States of America; 4 Department of Pharmacology and Toxicology, Geisel School of Medicine at Dartmouth, Hanover, New Hampshire, United States of America; University of Pittsburgh, United States of America

## Abstract

PDZ domains are protein-protein interaction modules that coordinate multiple signaling and trafficking pathways in the cell and that include active therapeutic targets for diseases such as cancer, cystic fibrosis, and addiction. Our previous work characterized a PDZ interaction that restricts the apical membrane half-life of the cystic fibrosis transmembrane conductance regulator (CFTR). Using iterative cycles of peptide-array and solution-binding analysis, we targeted the PDZ domain of the CFTR-Associated Ligand (CAL), and showed that an engineered peptide inhibitor rescues cell-surface expression of the most common CFTR disease mutation ΔF508. Here, we present a series of scaffolds containing chemically modifiable side chains at all non-motif positions along the CAL PDZ domain binding cleft. Concordant equilibrium dissociation constants were determined in parallel by fluorescence polarization, isothermal titration calorimetry, and surface plasmon resonance techniques, confirming robust affinity for each scaffold and revealing an enthalpically driven mode of inhibitor binding. Structural studies demonstrate a conserved binding mode for each peptide, opening the possibility of combinatorial modification. Finally, we diversified one of our peptide scaffolds with halogenated substituents that yielded modest increases in binding affinity. Overall, this work validates our approach and provides a stereochemical foundation for further CAL inhibitor design and screening.

## Introduction

One of the key goals of drug development is selectivity. Without it, side effects can overwhelm even highly promising therapeutic effects [1]. It has proven especially difficult to design selective inhibitors of a common class of targets, the protein-protein interaction PDZ domains, named after the founding members PSD-95, Dlg, and ZO-1 [2], [3], [4], [5]. PDZ domains generally recognize the extreme C-termini of target proteins, and engage only a limited set of motif residues [6], [7]. As a result, they frequently exhibit considerable overlap in their binding profiles. Furthermore, PDZ interactions are transient, and affinities are typically in the micromolar range [8]. Thus, despite over a decade of research, there are currently no reports of PDZ domain inhibitors in clinical trials or late stages of drug development.

In previous work, we have investigated the binding preferences of a PDZ domain that is known to interact with the C-terminus of the cystic fibrosis transmembrane conductance regulator (CFTR). The CFTR-associated ligand (CAL) controls the apical membrane half-life of CFTR and is a validated therapeutic target for the disease cystic fibrosis [5], [9], [10]. We have previously described a peptide array-based approach that enabled us to design a selective inhibitor for the CAL PDZ (CALP) domain. The result was iCAL36, a decameric peptide with sequence ANSRWPTSII [5], [9].

Despite this success, the micromolar CALP-binding affinity of iCAL36 remains weak by pharmacological standards. To address this limitation we sought a platform that would enable us to expand the chemical space available for inhibitor optimization. Given the dearth of high-affinity small-molecule PDZ inhibitors and the inherent propensity of such domains to bind peptides, we decided to focus on peptidomimetic strategies, which include the addition of lipids, chemical moieties, cycles, non-natural amino acids and backbone chemistries [2], [3], [4], [5], [11], [12]. Such modifications can increase the contact surface area of the interaction, and thus potentially enhance its affinity. In addition, since non-natural moieties can reach surfaces outside the peptide-binding cleft, these peptides may be more specific than those derived from naturally occurring amino acids [13], [14]. As a result, iterative rounds of stereochemical refinement may provide opportunities to develop higher affinity, more selective inhibitors.

Indeed, CALP provides an ideal system to investigate the biochemistry of chemically modified peptides. We know peptide inhibitors are efficacious in cells [5], [9], [15], and we can use X-ray crystallography to investigate the stereochemistry of the interactions at high resolution [16], [17]. We have previously explored dirhodium modification of CALP peptide inhibitors and seen a robust effect; however, crystallization efforts with those peptides have so far been unsuccessful due to solubility limits of the peptides [18]. Here, we describe another strategy, with the goal of testing the suitability of a set of iCAL36-based scaffolds, each carrying a lysine side-chain acceptor for chemical modifications at one of the non-motif positions along the peptide-binding cleft. In addition, we use structural and biochemical techniques to directly investigate the hypothesis that side-chain modification can regulate the affinity of peptide-based inhibitors for a PDZ target without disrupting the binding pose of the complex.

## Materials and Methods

### Protein and Peptide Synthesis

CALP protein was expressed and purified as previously described [19]. The peptides described in Table 1, with the exception of iCAL36 used in FP studies (which was synthesized by the Tufts Core Facility), were prepared at room temperature (RT) using standard Fmoc-based solid-phase peptide synthesis protocols, with modifications to accelerate preparation. Pre-loaded Fmoc-Ile-Wang resin was swollen 30 min in dimethylformamide (DMF), drained, deprotected with piperidine/DMF (shaken 1 min; drained; repeated once), and washed with DMF (shaken 15 sec; drained; repeated twice). During sequential coupling of the remaining residues, the appropriate Fmoc amino acid was precombined with peptide coupling reagent 2-(6-chloro-1H-benzotriazole-1-yl)-1,1,3,3-tetramethylaminium hexafluorophosphate (5 equivalents each/mol bound resin) in DMF and added to the resin. After mixing for 20 seconds, *N,N′*-diisopropylethylamine (DIEA) was added and allowed to react for 3 min, followed by an additional DMF wash (shaken 15 sec, drained; repeated twice). These steps were iterated for each standard amino acid. For modified residues, Lys with side chain 4-methyltrityl (Mtt) protection was incorporated at the appropriate position. Introduction of the desired organic acid was effected through selective Mtt removal (2% [*v/v*] trifluoroacetic acid [TFA] at RT for 1 h), after which the specific organic acid was coupled (*N,N′*-diisopropylcarbodiimide and DIEA in DMF, 30 min). Remaining residues were added using the conditions described above. After final Fmoc deprotection, the resin mixture was pipetted into a vessel, and the resin was sequentially washed with DMF and dichloromethane (twice each). Peptide removal from the resin and global deprotection were accomplished by addition of resin cleavage solution (5× resin volume of TFA and scavenger mixture triisopropylsilane/thioanisole/anisole [90∶4∶3∶3]), using microwave heating at 38°C for 30 minutes (Discover S-Class microwave synthesizer; CEM Corporation).

**Table 1:** Comparison of binding affinities measured by various techniques.

Peptide	Sequence	*K*_D_ from FP (µM)	*K*_D_ from ITC (µM)^a^	*K*_D_ from SPR (µM)^a^
iCAL36	ANSRWPTSII	22.6±8.0^b^	42.2±4.0	44.2±0.3
			(1.9)^c^	(2.0)^c^
Ac-iCAL36	Ac-ANSRWPTSII^d^	22.6±1.7	73.2±12.9	74.0±2.6
			(3.2)	(3.3)
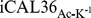	ANSRWPTS[Ac-K]I^e^	14.9±5.8	26.4±4.5	39.5±3.2
			(1.8)	(2.7)
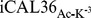	ANSRWP[Ac-K]SII^e^	180±110	91.9^f^	122±10
			(0.5)	(0.7)
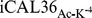	ANSRW[Ac-K]TSII^e^	32.3±6.2	49.4^f^	64.6±4.0
			(1.5)	(2.0)
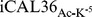	ANSR[Ac-K]PTSII^e^	550±400	ND	298±5
				(0.5)
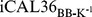	ANSRWPTS[BB-K]I^g^	11.1±3.9	20.7±2.0	20.0±0.4
			(1.9)	(1.8)
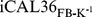	ANSRWPTS[FB-K]I^h^	11.4±3.7	12.8±1.7	27.9±3.6
			(1.1)	(2.4)
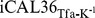	ANSRWPTS[Tfa-K]I^i^	14.1±2.0	34.5±8.8	22.3±4.2
			(2.4)	(1.6)

a *K*_D_ values as reported in ref. [20].

b Value previously reported in ref. [17].

c Value in parentheses indicates fold-deviation from the value obtained by FP.

d Ac- indicates N-terminal acetylation.

e [Ac-K] indicates N_ζ_-acetyl-lysine.

f n = 1.

g [BB-K] indicates N_ζ_-4-bromobenzoyl-lysine.

h [FB-K] indicates N_ζ_-4-fluorobenzoyl-lysine.

i [Tfa-K] indicates N_ζ_-trifluoroacetyl-lysine.

All peptides were purified using reverse-phase HPLC. Masses were confirmed by either liquid chromatography/mass spectrometry (Shimadzu LCMS-2020) or matrix-assisted laser desorption/ionization time-of-flight mass spectrometry (Voyager DE), and peptides were then lyophilized to white solids.

### Fluorescence Polarization (FP)

FP competition experiments were performed as previously described [16], [17]. The reporter peptide for CALP was F*-iCAL36 (fluorescein coupled via an amino-hexanoic acid linker to the N-terminus of ANSRWPTSII; *K*_D_ = 0.97 µM), and the CALP concentration was 1.5×*K*_D_. Experiments were performed in triplicate using separate protein-reporter and peptide stock solutions, and *K*_I_ values were determined using least-squares fitting of the set of competitive binding equilibria.

### Isothermal Titration Calorimetry (ITC)

ITC experiments were performed and analyzed in a manner similar to that described previously [14], [20]. Titrations were performed with a Nano ITC 2G (TA Instruments). In brief, for a typical titration, 100–320 µM CALP was loaded into the 943 µL sample cell, and 2–3 mM peptide placed in a 250 µL injector syringe. For 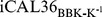, titrations were performed with polyhistidine-tagged CALP. Peptide concentrations were adjusted based on the amount of protein, so the final ratio of peptide to protein ranged between 3∶1–4∶1. Each titration included an initial 1 or 2 µL injection followed by 5 or 10 µL injections performed at 180 s intervals, for a total of 25–50 injections. The stirring speed was 250 rpm, and experimental temperature was 25°C. Thermograms were analyzed using NanoAnalyze software (TA Instruments). Experiments were performed in duplicate (n = 2), except as indicated in Table 1. Details are described in ref. [21].

### Surface Plasmon Resonance (SPR)

SPR experiments were performed on a Biacore X100 Plus (GE Healthcare Life Sciences), with CALP covalently immobilized on a CM5 sensor chip. Peptide solutions were prepared using HBS-EP buffer, and a series of dilutions from approximately 10×*K*_D_ to 0.1×*K*_D_. SPR experiments were conducted for each peptide dilution, and the equilibrium analysis mode of the instrument was used to determine the dissociation constant. Sensorgrams were analyzed using the Biacore evaluation software. Experiments were performed in duplicate or triplicate (n≥2). A detailed description is provided in ref. [21].

### Crystallization and Structure Determination

CALP was co-crystallized with each of the 7 side-chain modified peptides as previously described [16], [17], [19]. Crystallization conditions for all complexes were similar and are listed in Tables 2 and 3. Crystals appeared in 1–2 d and grew for up to 2 weeks before harvest and flash-cooling. Cryoprotectant solutions were also similar (35% [*w/v*] PEG 8000 or PEG 3350, 100–200 mM sodium chloride, 100 mM Tris pH 7.5, 20% [*w/v*] glycerol).

**Table 2:** Data collection and refinement statistics for iCAL36 acetylated peptide derivatives in complex with CALP.

	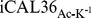	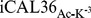	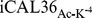	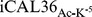
**Data Collection**				
Space Group	*P* 2_1_ 2_1_ 2_1_	*P* 2_1_ 2_1_ 2_1_	*P* 2_1_ 2_1_ 2_1_	*P* 2_1_ 2_1_ 2_1_
Unit cell dimensions				
*a,b,c* (Å)	36.3,47.7,97.8	35.9,47.7,97.2	36.2,47.8,98.1	36,47.5,100.5
Resolution^a^ (Å)	19.5-1.4 (1.52-1.4)	19.5-1.3 (1.37-1.3)	19.7-1.4 (1.46-1.4)	19.5-1.55 (1.6-1.55)
R*_sym_* b (%)	7.2 (55.7)	8.4 (67.2)	6.3 (59.4)	8.0 (50.9)
I/σ_I_	30.16 (4.83)	29.31 (4.93)	23.23 (3.73)	23.85 (3.73)
Completeness (%)	99.8 (99.2)	99.6 (98.7)	99.9 (100)	99.2 (96.6)
Crystallization conditions	37% (*w/v*) PEG 8000	34% (*w/v*) PEG 3350	40% (*w/v*) PEG 3350	31% (*w/v*) PEG 8000
	0.15 M NaCl	0.2 M NaCl	0.15 M NaCl	0.15 M NaCl
	0.1 M Tris pH 7.5	0.1 M Tris pH 7.5	0.1 M Tris pH 7.5	0.1 M Tris pH 7.5
**Refinement**				
Total # of reflections	34,123	41,732	32,997	25,711
Reflections in the test set	1,716	2,094	1,660	1,310
*R*_work_ c/*R*_free_ d	18.4/20.3	18.1/20.0	18.1/19.9	18.8/21.9
Number of atoms:				
Protein	1343	1367	1342	1314
Water	203	214	199	265
Ramachandran plot^e^ (%)	97.4/2.6/0/0	95.3/4.7/0/0	96.1/3.9/0/0	97.3/2.7/0/0
*B*_av_ (Å^2^)				
Protein	12.22	11.09	13.79	12.59
Solvent	24.92	22.36	24.24	24.80
Bond length RMSD (Å)	0.012	0.006	0.014	0.006
Bond angle RMSD (°)	1.221	1. 076	1.314	1.060

a Values in parentheses are for data in the highest-resolution shell.

b *R*_sym_ = Σ*_h_*Σ*_i_* |I(*h*)−I*_i_*(*h*)|/Σ*_h_*Σ*_i_* I*_i_*(*h*), where I*_i_*(*h*) and I(*h*) values are the *i-*th and mean measurements of the intensity of reflection *h*.

c *R*_work_ = Σ∥F_obs_|_h_−|F_calc_∥_h_/Σ |F_obs_|_h_, h ∈ {working set}.

d *R*_free_ is calculated as *R*_work_ for the reflections h ∈ {test set}.

e Core/allowed/generously allowed/disallowed.

**Table 3:** Data collection and refinement statistics for chemically-modified iCAL36 peptide derivatives, bound to CALP.

	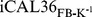	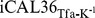	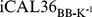
**Data Collection**			
Space Group	*P* 2_1_ 2_1_ 2_1_	*P* 2_1_ 2_1_ 2_1_	*P* 2_1_ 2_1_ 2_1_
Unit cell dimensions			
*a,b,c* (Å)	36.3,47.7,97.8	36.2,47.9,97.4	36.0,47.9,97.5
Resolution^a^ (Å)	19.5-1.7 (1.76-1.7)	19.5-1.4 (1.52-1.4)	19.5-1.4 (1.46-1.4)
R*_sym_* b (%)	8.9 (60.0)	7.6 (56.4)	7.8 (68.6)
I/σ_I_	16.33 (3.46)	17.6 (3.72)	25.63 (4.29)
Completeness (%)	99.9 (99.9)	96.3 (94.2)	99.6 (99.1)
Crystallization conditions	33% (*w/v*) PEG 3350	33% (*w/v*) PEG 3350	31% (*w/v*) PEG 3350
	0.2 M NaCl	0.2 M NaCl	0.1 M NaCl
	0.1 M Tris pH 7.5	0.1 M Tris pH 7.5	0.1 M Tris pH 7.5
**Refinement**			
Total # of reflections	19,345	32,898	33,913
Reflections in the test set	980	1,636	1,720
*R*_work_ c/*R*_free_ d	17.3/20.7	18.0/19.9	18.2/20.1
Number of atoms:			
Protein	1314	1368	1342
Water	181	228	204
Ramachandran plot^e^ (%)	96.7/3.3/0/0	96.7/3.3/0/0	96.7/3.3/0/0
*B*_av_ (Å^2^)			
Protein	14.94	12.15	11.96
Solvent	26.47	25.35	24.35
Bond length RMSD (Å)	0.007	0.0008	0.008
Bond angle RMSD (°)	1.100	1.129	1. 139

a Values in parentheses are for data in the highest-resolution shell.

b *R*_sym_ = Σ*_h_*Σ*_i_* |I(*h*)−I*_i_*(*h*)|/Σ*_h_*Σ*_i_* I*_i_*(*h*), where I*_i_*(*h*) and I(*h*) values are the *i-*th and mean measurements of the intensity of reflection *h*.

c *R*_work_ = Σ∥F_obs_|_h_−|F_calc_∥_h_/Σ |F_obs_|_h_, h ∈ {working set}.

d *R*_free_ is calculated as *R*_work_ for the reflections h ∈ {test set}.

e Core/allowed/generously allowed/disallowed.

Diffraction data were collected on beamline X6A at the National Synchrotron Light Source at λ = 0.8856 Å. The crystal-to-detector distance varied from 120–180 mm. 0.3° oscillation images were collected over a range of 360° for all crystals, with the exception of 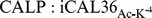 (180° range). Data were processed using the XDS and CCP4 programs, as previously described [16].

**Figure 1:**
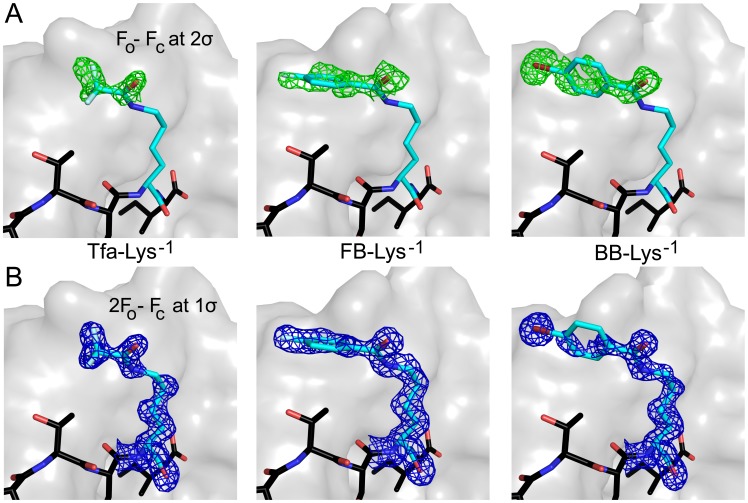
Electron density of chemically modified peptides. A) Prior to ligand modeling, positive electron density was observed in *F*_O_ – *F*_C_ difference maps, as illustrated here (contour level: 2σ) for the halogenated substituents attached to the Lys side chain at P^−1^. B) After iterative rounds of refinement and model building, final 2*F*_O_ – *F*_C_ electron density maps (contour level: 1σ) showed excellent agreement with the model, as shown here for the corresponding P^−1^ side chains. The final refined peptide structures are shown as stick figures and colored by atom/position (C = light blue for P^−1^ or black for other positions, O = blue, F = sky blue, N = red, Br = dark red). The van der Waals surface of CALP is shown in gray. The substituents are indicated as follows: Tfa, trifluoroacetic acid; FB, 4-fluorobenzoic acid; BB, 4-bromobenzoic acid.

Phases were calculated using molecular replacement in PHENIX [22], with the CAL PDZ domain as a search model (A-protomer of PDB entry 4E34). Since no ligand was included in the search model, the observation of clear electron density for the bound peptide was used to assess initial phase quality. Representative *F*_O_-*F*_C_ electron density starting ‘omit’ maps are shown in the panels in Figure 1A for each of the three halogenated moieties. Models were built from composite omit maps and refined using PHENIX. The electron density following refinement is shown for each of the three modified side chains in separate panels in Figure 1B. Structure geometries were validated using MOLPROBITY and the PDB Validation Server [23], [24]. PYMOL was used to render figures [25]. Electrostatic potential surface maps were determined using APBS [26]. AREAIMOL, part of the CCP4 suite of programs, was used to calculate solvent accessible surface area [27], [28]. Data collection and refinement statistics are reported in Tables 2 and 3. All structures have been deposited in the Protein Data Bank, with accession codes 4NMO, 4NMP, 4NMQ, 4NMR, 4NMS, 4NMT, and 4NMV.

## Results

### A set of scaffolds for chemical modification

**Figure 2:**
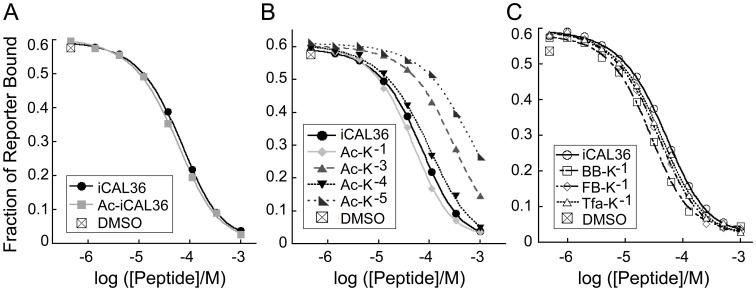
Fluorescence polarization binding studies of modified peptides. Representative FP isotherms are shown for the displacement of CALP:reporter binding by (A) Ac-iCAL36, (B) scaffold peptides containing acetylated lysine substitutions at each non-motif position, or (C) peptides containing halogenated substituents at the P^−1^ position (C). In each panel, iCAL36 displacement is also shown for reference. A fluoresceinated iCAL36 peptide (F*-iCAL36) was used as the reporter for all measurements. For illustrative purposes, a logistic curve-fit is shown, but *K*_I_ values and fractional reporter occupancy values were determined by least-squares fitting of the complete binding equation. Summary values of independent experiments (n = 3) are reported in Table 1.

As a first step in developing peptidomimetic acceptor scaffolds, we investigated the importance of the chemistry at the peptide N-terminus. Specifically, since native binding partners of the CAL PDZ domain are not short peptides, but rather intact proteins, we tested whether N-terminal acetylation (Ac-) would enhance binding affinity by neutralizing the charged peptide amino-terminal moiety at the P^−9^ position. In head-to-head fluorescence polarization (FP) displacement assays, Ac-iCAL36 (sequence: Ac-ANSRWPTSII) showed no significant change in binding affinity compared to iCAL36 (sequence: ANSRWPTSII; Figure 2A and Table 1). For subsequent experiments, we therefore used peptides with native N-termini.

Recent work from our lab revealed that positions along the entire length of the CALP binding cleft can contribute to peptide affinity [17]. We therefore considered substituting lysine residues at non-motif positions along the iCAL36 backbone, to provide primary amino groups for facile downstream modification. A previously published substitutional analysis of the iCAL36 sequence by peptide array [9], [17] suggested that replacement with lysine should yield baseline or enhanced affinity at the P^−1^ or P^−4^ positions, but weaker binding at the P^−3^ or P^−5^ positions. However, analysis of SubAna arrays based on alternative starting sequences suggested that lysine substitution at the P^−3^ and P^−5^ positions might not be detrimental [17]. In order to test which positions can accommodate a modified lysine residue without a substantial loss in peptide binding affinity, we substituted residues at the P^−1^, P^−3^, P^−4^, and P^−5^ positions of the iCAL36 peptide. Each substituted lysine was capped with an acetyl group, to more closely mimic the electrostatic character that would be present following modification with an organic acid (here: acetic acid).

The sequences of the resulting peptides 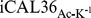, 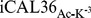, 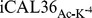, and 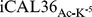, are listed in Table 1, along with binding affinities determined by FP displacement assays (Figure 2B). Consistent with our SubAna data, modifications at the P^−1^ and P^−4^ positions yielded the highest affinities, comparable to or slightly better than the affinity of the template sequence. Substitution of an acetylated lysine at the P^−3^ and P^−5^ positions yielded binding constants weaker by ∼8-fold and ∼24-fold, respectively. Thus, while each of the substituted peptides retained binding affinity, different positions exhibited differential sensitivity, consistent with our previous evaluation of ‘modulator’ stereochemical preferences at non-motif positions [17].

### Structural analysis of acetyl-lysine scaffolds along CALP peptide binding cleft

**Figure 3:**
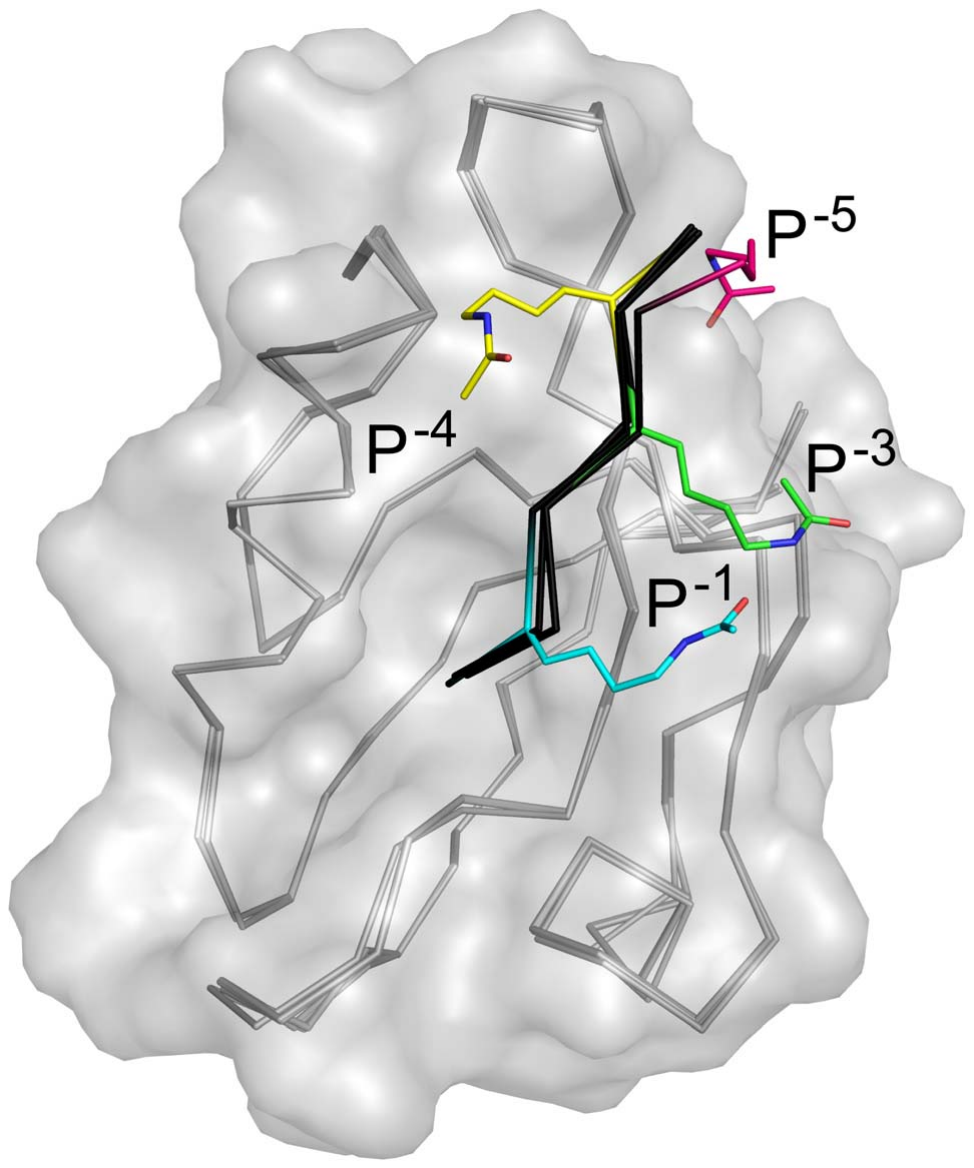
Chemically modifiable scaffolds at each non-motif position in the CALP binding cleft. The C_α_ trace (gray) shows all CALP structures, aligned by main chain atoms (RMSD ≤0.3 Å compared to CALP:iCAL36, PDB ID: 4E34). CALP is also shown as a transparent surface (gray). The peptides are shown as black C_α_ traces, with the acetylated lysine residue of each respective peptide highlighted (carbon atoms colored by position, as indicated). Peptide positions are labeled, and non-carbon atoms are colored separately: O = blue, N = red.

We next wanted to test the hypothesis that the lysine substitutions were accommodated without significant changes to the backbone geometry. The structures of CALP bound to each of the substituted peptides were determined by X-ray crystallography, for comparison with the structure in complex with iCAL36 [16]. Overall data collection and refinement statistics are reported in Table 2. As in previous analyses, co-crystals contained two protomers in the asymmetric unit. The A- and B-protomers are superimposable for all crystals, except for the 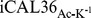 complex, in which the P^−1^ side-chain amino group is oriented towards solvent in protomer A, but forms a hydrogen bond with CALP-Ser^302^ in protomer B (distance: 2.9 Å), suggesting a dynamic interaction. In the following descriptions, we will use the B-protomers of each complex for our analyses. The refined structures reveal similar peptide conformations in the binding cleft (Figure 3). When compared to the CALP:iCAL36 structure, the root mean squared deviation (RMSD) for the main chain atoms in the B-protomers was ≤0.1 Å in all cases, except 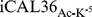 (RMSD = 0.3 Å).

**Figure 4:**
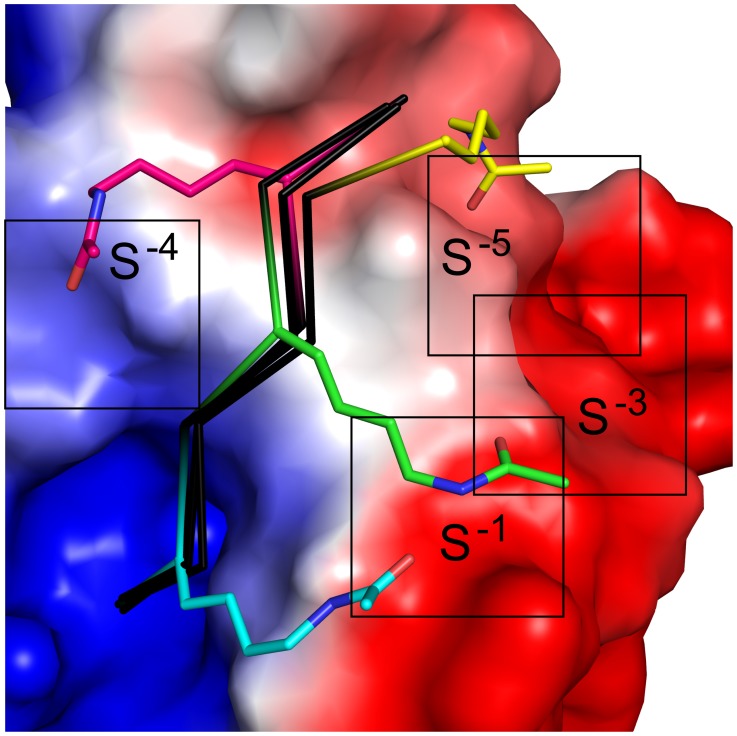
Differential stereochemical and electrostatic environments at each modifiable position. The electrostatic potential surface map of CALP is shown, rendered at ±10 k_B_T/e (negative surface in red, positive surface in blue). Boxes and labels (S^−1^, S^−2^, etc.) indicate an approximate area that chemical modifications could bind when attached to the lysine N_ζ_ moiety at that position. Overlap in accessible area suggests a possible region for side-chain cyclization. Peptide backbones are shown as black C_α_ traces, with acetyl-lysine residues shown as sticks and colored by position. Non-carbon atoms are colored separately: O = blue, N = red.

We also wished to assess the stereochemical environment surrounding the terminal acetyl moiety at each position. Thus, although an acetylated lysine substitution is slightly more disruptive at P^−3^ than at P^−1^, there may be hydrophobic pocket accessible from P^−3^, for example, which could represent a target for further peptidomimetic engineering. Indeed, alignment of the structures of our acetylated complexes reveals different stereochemical environments for each substituted position. For example, a P^−4^ acetylated lysine interacts with the CALP α2 helix, whereas a P^−1^ or P^−3^ acetylated lysine faces the opposite side of the peptide binding cleft, and a P^−5^ acetylated lysine can access either side of the interaction pocket (Figure 3). Furthermore, the electrostatic potential surface of CALP reveals that the site interacting with the P^−4^ acetylated lysine residue (S^−4^) is positive. The other three sites are negative, and S^−1^ and S^−3^ are more negative than S^−5^ (Figure 4). Taken together, all four positions are potential sites for targeted differential modification.

### Chemically-modified peptides increase binding affinity for CALP

**Figure 5:**
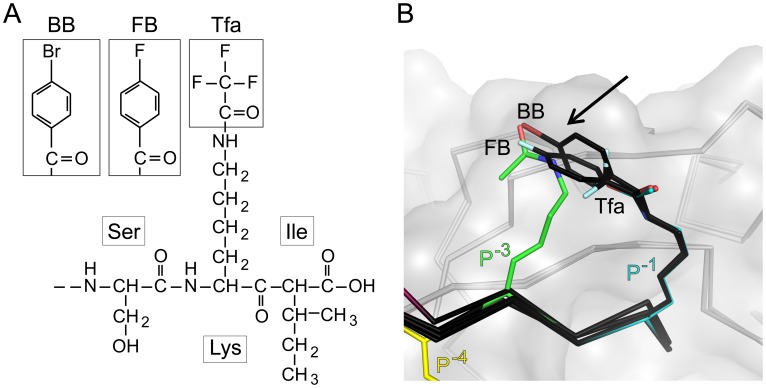
Chemical modifications at the P^−1^ position reveal similar modes of binding to CALP. A) Schematic representations of the three additional chemically modified peptides are shown for reference and labeled by modification: 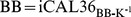, 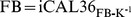, 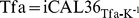. B) The structures of CALP (gray C_α_ trace and van der Waals surface) in complex with these peptides (stick figures, black carbons), or in complex with peptides bearing acetylated lysines at P^−1^ (blue carbons), P^−3^ (green carbons), and P^−4^ (yellow carbons) are shown following alignment. Superposition of these structures reveals that all halogenated substituents interact with a similar region (arrow) at the edge of the CALP peptide-binding cleft, in close proximity to the P^−3^ binding site. Non-carbon atoms are colored by element: O = blue, F = sky blue, Br = dark red, N = red.

For proof-of-principle studies, we chose to further diversify the position with the highest baseline affinity, i.e., P^−1^ in 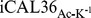, by introducing three halogenated substituents. Halogenated organic moieties are found in a disproportionate number of biologically active substances of both natural and synthetic origin, and halogenated aromatic residues can significantly contribute to binding affinity [29]. Further, hundreds of aromatic organic acids, halogenated and otherwise, are commercially available. Thus, our initial set of ligands enables us to test the feasibility of affinity modification by preparing and screening a larger library of chemically-modified peptides [14]. At the P^−1^ position, we coupled trifluoroacetic acid (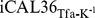), 4-fluorobenzoic acid (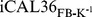), or 4-bromobenzoic acid (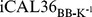) to the side-chain terminal amino group, as illustrated in Figure 5A. The binding affinities of the resulting peptides were determined by FP displacement (Figure 2C) and are listed in Table 1. All three peptides reveal modestly enhanced binding affinity. Overall, the largest increase is for 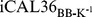 and 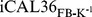, which are each ∼2-fold stronger than iCAL36.

To explore the stereochemistry of these interactions, we also solved the complex structures of CALP bound to each of these three peptides. All data collection and refinement statistics are in Table 3. The modified lysine and the terminal moieties are oriented similarly in the complexes with 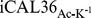 and with the additional P^−1^ substituted peptides (Figures 1B and 5B). Furthermore, the distal functional groups overlap with the surface on CALP contacted by the acetyl group of the P^−3^ lysine in 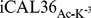 (Figure 5B). Thus, S^−1^ and S^−3^ can intersect and could potentially be bridged using a cyclic peptide, a strategy previously employed in targeting PSD-95 [11].


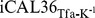
, 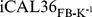, and 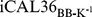 bind CALP with affinities modestly higher than the value observed for iCAL36 (Table 1). In addition, the substituents increase the contact surface area of the modified P^−1^ lysine residue by 69, 32, and 40 Å^2^, respectively. Finally, they can serve as chemical scaffolds for additional elaboration. Indeed, the availability of high-resolution stereochemical information for each of the complexes with CALP (Figure 5B) will facilitate *in silico* docking screens to identify candidates for more detailed biochemical and structural characterization.

### Comparison of binding constants determined by multiple techniques

There are a number of techniques used to measure PDZ domain:peptide binding affinities [as discussed, e.g., in ref. 20]. In some cases, significant technique-dependent differences have been reported for PDZ ligands (e.g., [30], [31]). Here, we directly compare the three most prevalent techniques and investigate the affinities of these singly substituted peptides, as well as to an unmodified iCAL36 peptide, in parallel by FP, ITC, and SPR (Table 1). Across techniques, the rank order of affinities is largely conserved. The P^−1^-substituted peptides are the four highest-affinity ligands, and rank-order switches are found largely within experimental uncertainties. Relative to iCAL36, there is little change in affinity for the P^−4^-substituted peptide, and the P^−3^- and P^−5^-substituted peptides are the weakest ligands. The most significant rank-order reversal involves the Ac-iCAL36 peptide, for which the ITC and SPR values are ∼3-fold larger than the FP value. Indeed, with the exception of the two weakest ligands with larger uncertainties, the SPR values are generally 2–3-fold higher than the FP values. The ITC values are somewhat more variable, but mostly fall at or between the FP and SPR values (Table 1). Overall, despite modest differences, the results are largely concordant, with the highest affinities seen for 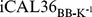 (FP and SPR) and 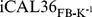 (ITC).

## Discussion

As regulators of important intracellular trafficking and scaffolding processes, PDZ domains are potentially attractive therapeutic targets [32]. However, despite promising new approaches such as fragment-based design, attempts to develop small-molecule inhibitors have so far faced limits of druggability common to protein-protein interactions [33]. High-affinity (*K_D_*≤20 nM) peptides have been engineered, which could serve as competitive inhibitors [34], [35]. However, as therapeutics, native peptides are susceptible to proteolysis and often have low inherent membrane permeability, which has led to an increasing interest in peptidomimetic strategies [36]–[39]. Here, we have tested the feasibility of developing competitive inhibitors of the CAL PDZ domain by chemically modifying a series of peptides carrying lysine acceptor residues at non-motif positions.

A limited set of four organic acid modifications of the iCAL36 P^−1^ side chain revealed modest enhancements of affinity. In evaluating these inhibitor leads, we performed a direct comparison of estimates of PDZ domain binding affinities determined by ITC, SPR, and FP. For specific peptide:PDZ complexes, the precise values showed some variation between binding methods. Since all three methods were performed under equilibrium binding conditions, it is unclear what caused the ∼3-fold discrepancy in *K_D_* values observed, e.g. for the N-terminally acetylated iCAL36 peptide (Table 1). Each technique does involve different incubation times and temperatures, as well as the presence or absence of stirring, shaking, or laminar flow, suggesting the need for care in evaluating the significance of subtle affinity shifts, both within and across techniques. Nevertheless, the ranking of strong, modest, and weak binders was concordant across techniques, suggesting that each method can each be reliably used to monitor changes in affinity substantial enough to affect ultimate *in vivo* therapeutic potency.

The approach described here is similar to the strategy used in the design of chemically modified peptides to inhibit both the PDZ3 domain of postsynaptic density protein 95 (PSD-95) for the treatment of stroke, and the PDZ domain of the GAIP-interacting protein, C-terminus (GIPC) for cancer therapy [13], [14]. In the PSD-95 PDZ3 study, binding affinities for the chemically modified peptides were measured, and the stereochemistry of binding was analyzed using NMR footprinting [13]. For GIPC inhibitors, cellular efficacy was confirmed, although data were not available for binding affinities or complex stereochemistry [14]. Our investigation of chemically modified peptide binding to CALP combines both thermodynamic and crystallographic evaluation of a series of chemically modified peptide scaffolds binding to a shared PDZ domain target.

In particular, our structural studies confirm that even modified peptides bind to the CAL PDZ domain in a common conformation, such that each peptide side chain interacts with a defined stereochemical environment [17]. As a result, our acetylated lysine peptide structures provide templates for future screening efforts. They can also be aligned to previously determined CAL PDZ domain structures. Thus, together with our previous studies of natural and metallated CALP inhibitor peptides [5], [9], [15], [18], these data help to establish an extensive framework for future peptidomimetic development. By combining multiple modifications to an already refined starting peptide sequence, we may achieve the chemical complexity necessary for truly selective CALP inhibition. If successful, such studies will provide additional proof-of-principle data for targeting of PDZ trafficking pathways in cystic fibrosis, as well as stereochemical leads to support the ultimate goal of therapeutic design.
